# Supplementary figures and images for: Breeding Tomato Hybrids for Flavour: Comparison of GWAS Results Obtained on Lines and F1 Hybrids
Source: Genes (Basel). 2021 Sep 18;12(9):1443. doi: 10.3390/genes12091443 (PMC8469758; doi:10.3390/genes12091443)

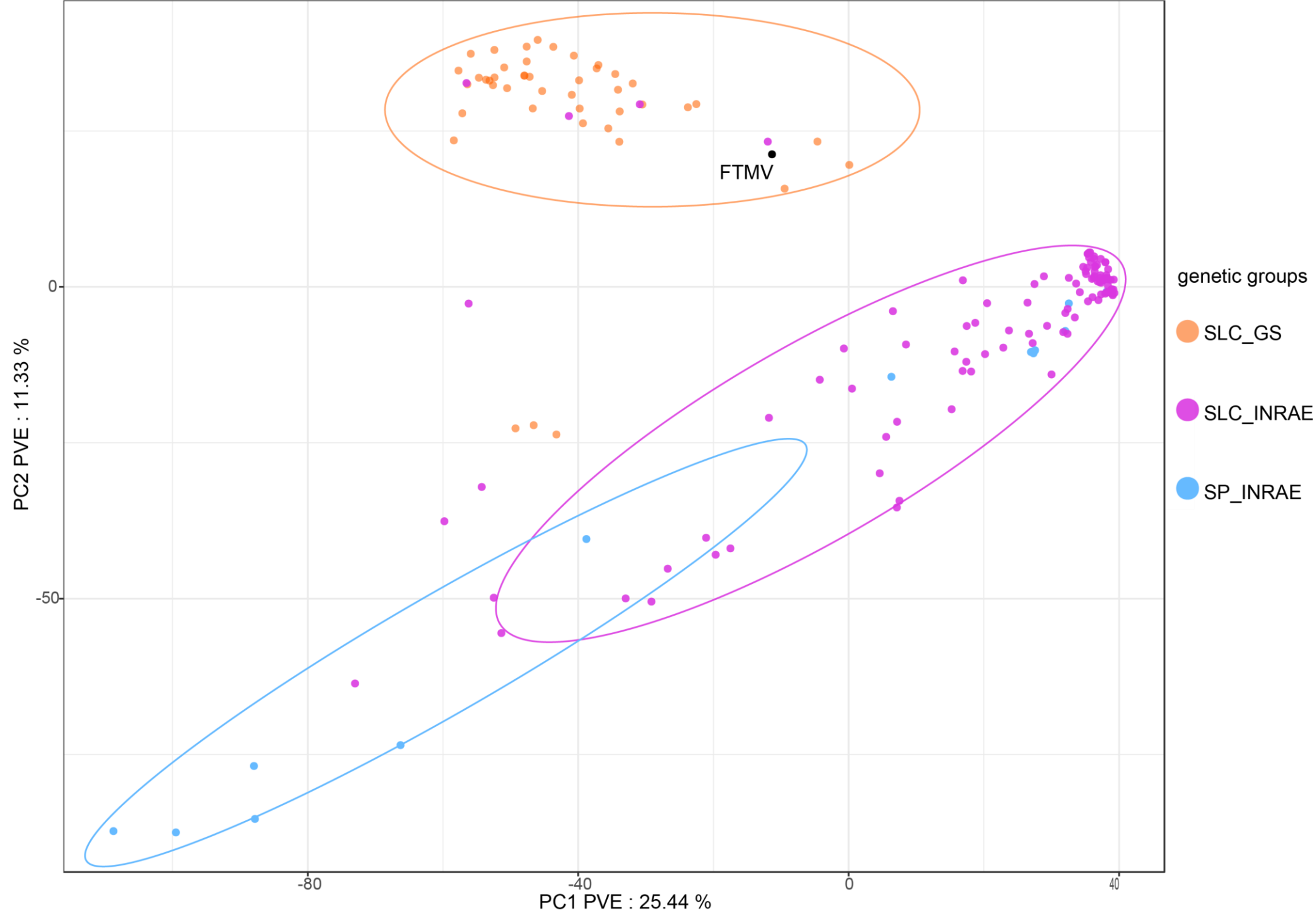

Samples

Metabolites

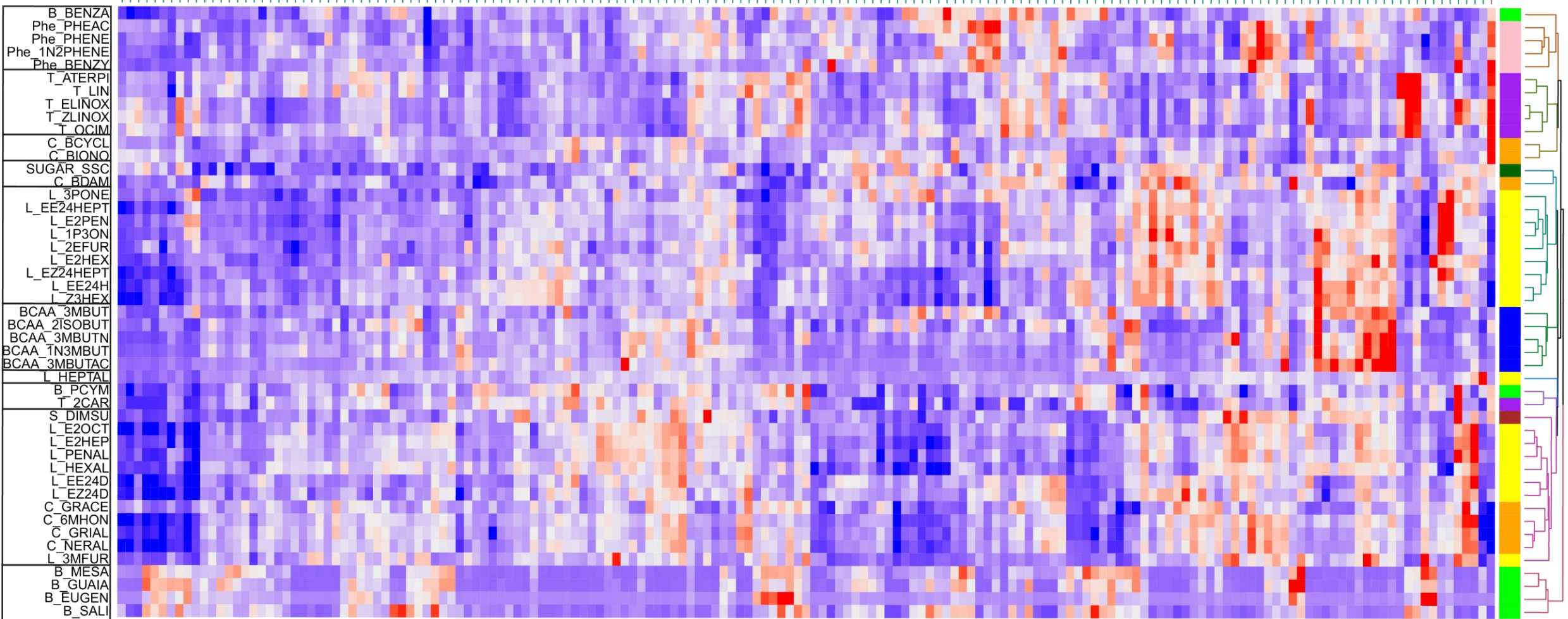

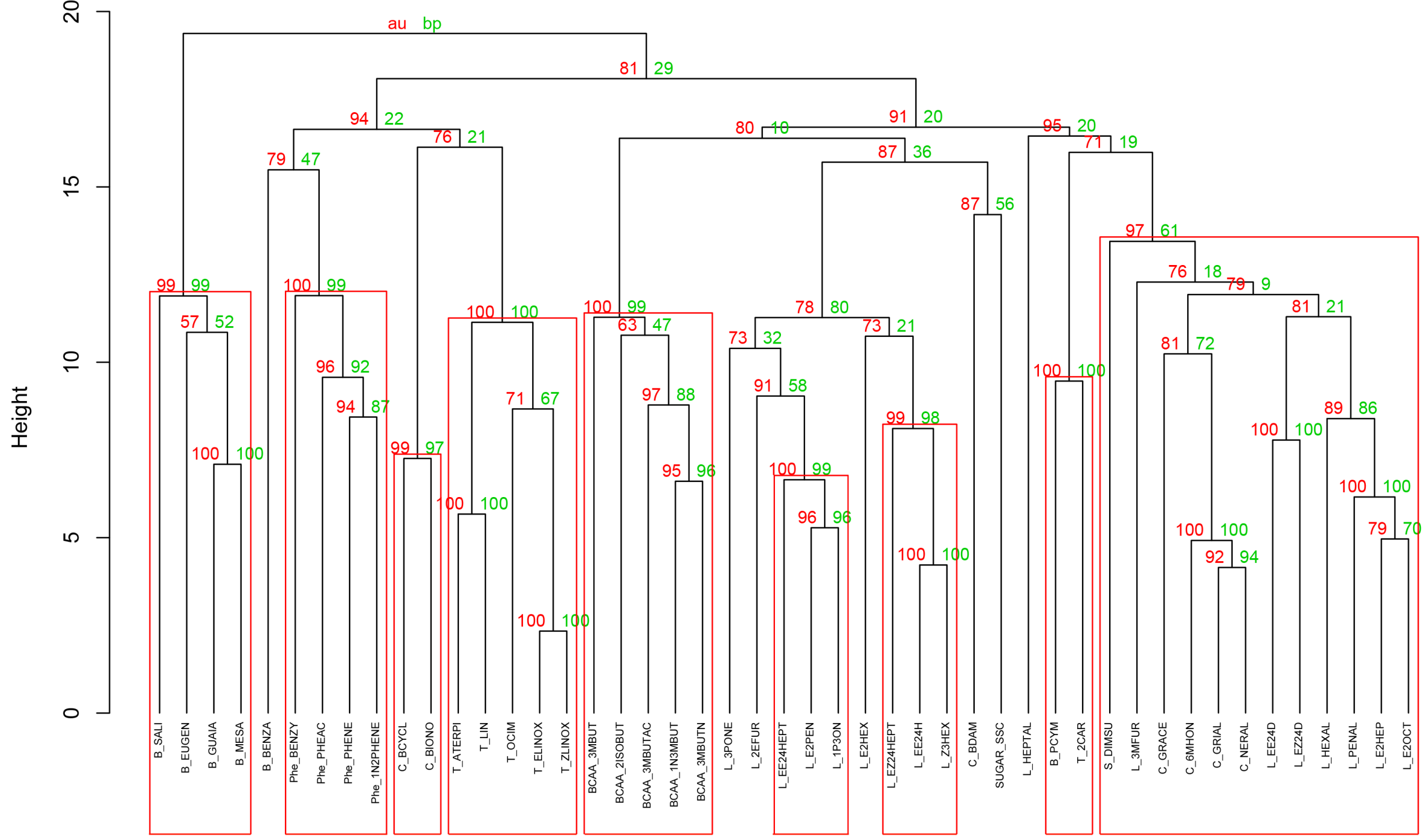

a)

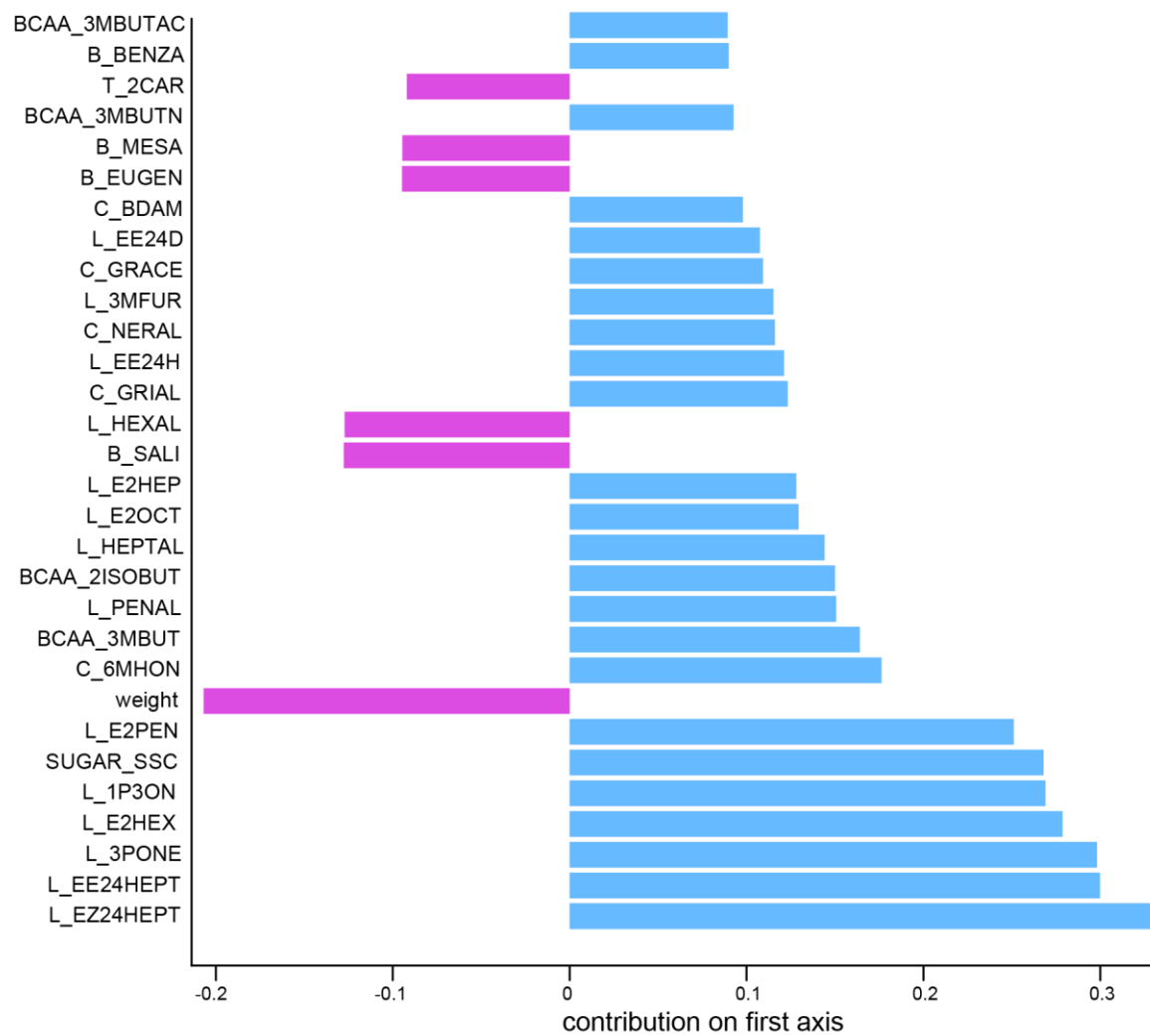

b)

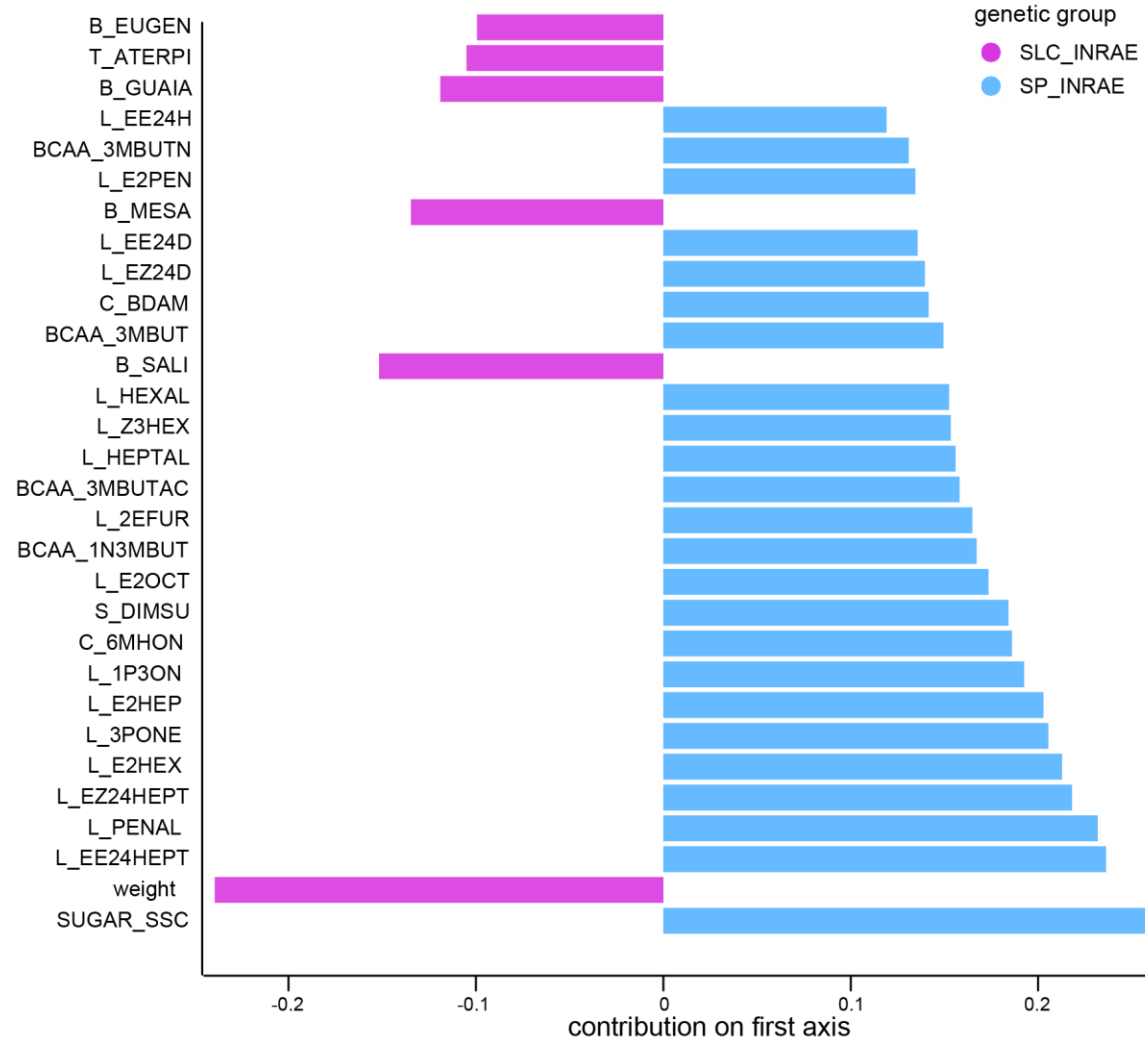

Supplement: Supplementary file 1 [file genes-12-01443-s001.zip › supp/Sup.Figures.pdf]
